# Absence of EpCAM in cervical cancer cells is involved in slug induced epithelial-mesenchymal transition

**DOI:** 10.1186/s12935-021-01858-3

**Published:** 2021-03-10

**Authors:** Xian Liu, Qian Feng, Yanru Zhang, PengSheng Zheng, Nan Cui

**Affiliations:** 1 Department of Reproductive Medicine, The First Affiliated Hospital of the Medical College, Xi’an Jiaotong University, 76 West Yanta Road, Shaanxi Province 710061 Xi’an, People’s Republic of China; 2 Section of Cancer Stem Cell Research, Key Laboratory of Environment and Genes Related to Diseases, Ministry of Education of the People’s Republic of China, Shaanxi 710061 Xi’an, People’s Republic of China

**Keywords:** Slug, EpCAM, EMT, Cervical cancer, Metastasis

## Abstract

**Background:**

Slug (Snai2) is a pivotal player in initiating epithelial-mesenchymal transition (EMT) through its trans-suppression effect on E-cadherin in various normal and malignant cells. In this study, the positive effect of Slug on promoting cell motility and metastasis in cervical cancer was further confirmed in this study.

**Methods:**

RNA-Seq was performed to explore the potential molecules that participate in Slug-mediated EMT in cervical cancer cells. The negative correlation between Slug and EpCAM expression in cervical cancer cells was detected in this study, and linked them with *in vitro* migration and invasion assay, *in vivo* metastasis experiments, luciferase reporter assay and Chromatin immunoprecipitation.

**Results:**

Transcriptome sequencing analysis revealed that *epithelial cell adhesion molecule (EpCAM)* was significantly decreased in Slug-overexpressing SiHa cells. Simultaneously, an absence of EpCAM expression was observed in Slug-overexpressing cells. Further studies revealed the trans-suppression effect of Slug on EpCAM through its binding to the E-boxes in the proximal promoter region of EpCAM in cervical cancer cells. Restoring EpCAM in Slug-overexpressing cells by transiently transfecting an EpCAM recombinant plasmid attenuated cell motility and promoted cell growth. Moreover, the negative correlation between Slug and EpCAM expression in human squamous cervical carcinoma (SCC) samples was verified by using Pearson correlation analysis.

**Conclusions:**

These results demonstrated that the absence of EpCAM under Slug expression in cervical cancer cells probably participated in Slug-regulated EMT and further promoted tumor metastasis. Additionally, this study supports a potential way for Slug to initiate EMT progression in cervical cancer cells in addition to inhibiting E-cadherin.

**Supplementary Information:**

The online version contains supplementary material available at 10.1186/s12935-021-01858-3.

## Background

Slug (Snai2), a member of the snail zinc-finger transcription factor family, has been reported to be pivotal player in initiating epithelial-mesenchymal transition (EMT) in various normal and malignant cells [1]. On a molecular level, Slug was reported to repress E-cadherin expression by binding special DNA sequences (E-box: CANNTG) in the proximal promoter region of the E-cadherin gene [2]. The absence of E-cadherin further induces the loss of tight junctions between epithelial cells, which is a key event for the initiation of EMT [3]. When epithelial cancer cells undergo EMT, polarized epithelial cells acquire invasive and migratory characteristics, which then facilitate cancer cell motility and distant metastasis [3]. This positive role of Slug in promoting EMT during tumor progression has been extensively researched in various types of cancers. As reported, silencing NatD in lung cancer cells suppresses EMT by downregulating Slug [1]. Death domain-associated protein (Daxx) binds E-boxes to antagonize the trans-suppressive effect of Slug on E-cadherin, subsequently stabilizing E-cadherin expression and suppressing cancer cell invasion and metastasis during hypoxia [4]. Overexpression of ubiquitin-specific protease 5 (USP5) promotes Slug stability and EMT in hepatocellular carcinoma [5]. Moreover, Slug was also reported to initiate EMT and promote metastasis through its trans-repression effect on E-cadherin regulation in cervical cancer [6, 7]. In addition, the trans-repression of E-cadherin and promotion of cell motility were confirmed in Slug-overexpressing cervical cancer cells in this study. However, in this study, exogenously expressed Slug in HeLa cells still has the capacity to enhance cell migratory and invasive abilities *in vitro*, despite E-cadherin being expressed at a very low protein level in HeLa cells. Therefore, whether there are other potential factors (besides E-cadherin) that are regulated by Slug and further initiate cell EMT is a very attractive research topic.

Epithelial cell adhesion molecule (EpCAM) is a transmembrane glycoprotein that was discovered in colorectal cancer cells 40 years ago [8]. The expression of EpCAM has been further demonstrated in numerous human normal epithelial tissues and carcinomas [9]. As EpCAM has “CAM” (cell adhesion molecule) properties, EpCAM was found to interact with other EpCAM molecules on neighboring cells in a homophilic interaction manner. Exogenous expression of EpCAM brings neighboring cells in close proximity, suggesting that EpCAM participates in cell adhesion [10]. Furthermore, EpCAM has also been reported to have adhesive properties (such as those of adherens junctions, tight junctions, hemidesmosomes and desmosomes) by interacting with other CAMs [11, 12]. In human carcinomas, EpCAM is commonly elevated in various primary tumor types. The high expression of EpCAM was shown to facilitate cancer malignant growth and progression and was associated with poor prognosis and therapeutic irresponsiveness in cancer patients [10, 13]. To complicate matters further, accumulating evidence has demonstrated that EpCAM likely has biphasic effects on regulating EMT, either enhancing or attenuating. On the one hand, EpCAM expression is decreased when cancer cells undergo EMT. In breast and lung cancer cells, a transient loss of EpCAM could be observed when cells undergo EMT during the metastasis process [14, 15]. Additionally, epithelial cancer cell lines exhibited decreased expression of EpCAM when cells were treated with TGF-β1 (transforming growth factor-β1) and TNFα (tumor necrosis factor-α), a combination that is known to induce EMT [16]. On the other hand, the promotion of EMT by EpCAM has also been reported in some studies. The expression of EMT-related transcription factors (e.g., Snail and Slug) was found to be inhibited in EpCAM knockdown colon cancer cells [17]. Elevated EpCAM expression could enhance TGF-β1-induced EMT in MCF-7 breast cancer cells [18]. Therefore, the results on the effect of EpCAM expression on cell migration and invasion are also conflicting in cancer cell lines, and this seems intrinsically linked with the biphasic effects of EpCAM on cell EMT regulation [13]. These contradictory observations on the role of EpCAM in mediating cell EMT probably suggest that there might be a window or stage that requires different functions of EpCAM during the process of cancer cells undergoing EMT and further participating in the metastasis process. EpCAM expression is probably dynamically regulated during EMT [10, 16].

In cervical cancer, the aberrant expression of EpCAM has been reported [19], and the presence of EpCAM in cervical adenosquamous carcinoma (ASC) is related to radiosensitivity [20]. In this study, exogenous expression of Slug in cervical cancer cell lines enhanced cell motility and further promoted distant metastasis. Simultaneously, a loss of EpCAM expression was observed in Slug-overexpressing cervical cancer cell lines. Further studies revealed the trans-suppression effect of Slug through its binding to the proximal promoter region of EpCAM to inhibit the expression of EpCAM in cervical cancer cells. These results support a potential alternative mechanism by which Slug promotes cell EMT in cervical cancer by trans-repressing EpCAM expression.

## Methods

### Cell culture

The human cervical cancer cell lines (HeLa, SiHa and CaSki) were purchased from American Type Culture Collection (ATCC; Manassas, VA) and were tested using RT-PCR for mycoplasma contamination every 3 months. The cell lines purchased from ATCC have been authenticated by STR profiling, so authentications were not performed.

Slug stably overexpression (SiHa and HeLa) and knockdown (CaSki) cell lines were generated as described in our previous study [21]. SiHa and HeLa cells were cultured in high-glucose Dulbecco’s modified Eagle’s medium (DMEM, Sigma-Aldrich, St Louis, MO, USA) with G418 (Calbiochem, La Jolla, CA, USA), CaSki cells were cultured in RPMI-1640 medium (Sigma-Aldrich, St Louis, MO, USA) with G418. And the culture media was contained 10% FBS (fetal bovine serum, HyClone, Thermo Scientific, Waltham, MA, USA).

### Plasmid construction and transfection

The pIRES2-EpCAM plasmid was constructed by using the following primers: forward, 5′-ACTAGCTAGCTAGCGCGCGCAGCATG-3′, and reverse, 5′-CACACGCGTCGACGTATGTACAAGACTC-3′. The primers were used to amplify the full-length human EpCAM coding sequence, which was cloned into the pIRES2-AcGFP vector (Clontech, Mountain View, CA) via the Nhe I and Sac I sites. Then, the plasmid was transiently transfected into Slug-overexpressing SiHa and HeLa cells by using Lipofectamine 2000 reagent (Invitrogen, Carlsbad, CA, USA). Cell growth curves and MTT assays were performed to detect cell proliferation in these cells, and migration and invasion assays were performed to detect cell motility.

### Immunocytochemistry and immunofluorescence assays

For the immunocytochemistry assay, SiHa-Vec and SiHa-Slug cells were seeded onto cover slips for 48 h, washed with PBS three times, fixed with 4% paraformaldehyde for 20 min, and permeabilized with 0.1% Triton X-100 for 20 min at room temperature. The cover slips were incubated with anti-EpCAM (1:200 dilution, sc-25,308, Santa Cruz, USA) and anti-Slug (1:50 dilution, #9585, Cell Signaling Technology, USA) at 4 °C overnight, and a horseradish peroxidase-conjugated secondary antibody was added for 30 min at room temperature.

For the immunofluorescence assay, after incubation with anti-EpCAM (1:50 dilution, sc-25,308, Santa Cruz, USA) and anti-Slug (1:50 dilution, #9585, Cell Signaling Technology, USA) overnight at 4 °C and washing with PBS, the cover slips were incubated with fluorescently conjugated secondary antibodies (Alexa Fluor 555, #A-31,570 and Alexa Fluor 488, # A-21,206, Thermo Fisher Scientific, USA) for approximately 60 min at room temperature. Finally, the cells were washed with PBS and incubated with DAPI (Solarbio). Cell images were taken with a laser scanning confocal microscope (Leica). The cells were imaged with an Olympus CX31 microscope digital camera and Leica DFC 500 digital camera and processed with ImageJ software.

### Immunohistochemistry assay

The human squamous cervical carcinoma (SCC) samples used in this study were collected at the First Affiliated Hospital of Xi’an Jiaotong University from 2008 to 2016 as described in our previous study [21]. The mouse xenografted tumor tissues derived from SiHa-Vec and SiHa-Slug cells were collected in our previous study, and the immunohistochemical staining procedure was performed as previously described [21]. The percentage of Slug or EpCAM positive cells was divided into 5 scores: <5% (0), 5–25% (1), 25–50% (2), 50–75% (3), and > 75% (4). The intensity of staining Slug or EpCAM was divided into 4 scores: no staining (0), light brown (1), brown (2), and dark brown (3). The immunohistochemistry (IHC) score of Slug and EpCAM staining was determined using the following formula: immunohistochemistry (IHC) score = percentage score × intensity score.

To validate the correlation between Slug and EpCAM expression *in vivo*, serial sections of human squamous cervical carcinoma samples (n = 15) were immunostained with an anti-EpCAM (1:200 dilution, sc-25,308, Santa Cruz, USA) and anti-Slug (1:50 dilution, #9585, Cell Signaling Technology, USA) antibody. The immunohistochemistry (IHC) score of Slug and EpCAM in these SCC samples was confirmed by using Pearson correlation analysis. The evaluation standard of immunohistochemistry (IHC) score was performed as previously described [21].

### Western blotting

The western blotting analysis used in this study was performed as previously described [21]. Horseradish peroxidase-conjugated anti-rabbit and anti-mouse IgG were purchased from Thermo Fisher Scientific (New York, NY, USA). The antibodies used for western blotting were as follows: anti-EpCAM (1:500 dilution, sc-25,308, Santa Cruz, USA), anti-β-catenin (1:500 dilution, sc-7963, Santa Cruz, USA), anti-cyclin D1 (1:500 dilution, sc-8396, Santa Cruz, USA), anti-GAPDH (1:500 dilution, sc-47,724, Santa Cruz, USA), and anti-Slug (1:1000 dilution, #9585, Cell Signaling Technology, USA). GAPDH was used as the control and for quantification.

#### ***In vitro*migration assay**

For the wound-healing assay *in vitro*, cervical cancer cells with stable Slug overexpression (SiHa and HeLa) or knockdown (CaSki) were plated in 6-well plates and scratched by using a pipette tip when the cells grew to nearly 100 % confluence. Wound images were taken at 0, 24 h and 48 h. The wound area was measured using ImageJ software, and the migration potential was calculated according to the equation: wound scratch area = (wound scratch area at 0 h) − (wound scratch area at 48 or 72 h). Two independent experiments were performed.

For the migration assay *in vitro*, 5 × 10^4^ cervical cancer cells with stable Slug overexpression (SiHa and HeLa) or knockdown (CaSki) were added to the top wells of transwell chambers that contained 1 % FBS (BD Biosciences, San Jose, CA) and were incubated for 48 h, while the bottom wells of the chambers contained 10 % FBS. The cells that migrated through the filter membrane were permeabilized with 70 % methanol and stained with 0.1 % crystal violet. The cells were imaged and counted under a microscope.

#### ***In vitro*invasion assay**

For the invasion assay *in vitro*, cervical cancer cells (10 × 10^4^ cells) with stable Slug overexpression (SiHa and HeLa) or knockdown (CaSki) were added to the top wells of transwell chambers that were coated with Matrigel and contained 1 % FBS and were incubated for 48 h. The bottom wells of the chambers contained 10% FBS. Then, the cells that migrated through the filter membrane were permeabilized with 70% methanol, stained with 0.1% crystal violet and counted under a microscope.

#### ***In vitro*cell growth assays**

After transiently transfecting a pIRES2-EpCAM plasmid into Slug-overexpressing SiHa and HeLa cells by using Lipofectamine 2000 reagent, cell growth curves and MTT assays were performed to detect cell proliferation in these cells. For cell growth curves, (4 × 10^4^) cells were seeded in 6-well plates in triplicate and counted on days 1, 3 and 5 by using a hemocytometer. For cell viability assays, cells were plated at a density of 800 cells per well and assessed by using 3-(4,5-dimethylthiazol-2-yl)-2,5-diphenyl tetrazolium bromide (Sigma-Aldrich, St Louis, MO, USA) dye, and the absorbance value at 490 nm was detected by using a plate reader at 1, 3 and 5 days.

#### ***In vivo*metastasis experiments**

The experimental protocols were evaluated and approved by the Animal Care and Use Committee of the Medical School of Xi’an Jiaotong University, and all of the animals were raised in a specific pathogen-free (SFP) environment with constant temperature (22–25 °C) and humidity (40–50%). For the *in vivo* metastasis experiments, ten female BALB/c-nude mice were randomly divided into two groups. SiHa-Vec and SiHa-Slug cells (6 × 10^5^) were injected into female nude mice via the tail vein (6- to 7-week-old female BALB/c-nude mice were purchased from SLAC Laboratory Animal Co., Ltd., Shanghai, China). At the end of the experiment (approximately two to three months), the mice were euthanized with carbon dioxide, and the lungs and livers were removed and subjected to histologic examination.

### Real‐time PCR analysis

Total RNA extraction and the protocol for real-time PCR were performed as previously described [21]. GAPDH was used as the housekeeping gene in this study, and all of the results were analyzed via the ∆∆Ct method. The primer sequences used in this study for real-time PCR were as follows: FN1 (F: 5′-AATCGTCAATGCCAGTGTACTT-3′ R: 5′-TCTCATCGCAGTCAGGATCATAA-3′) and GAPDH (F: 5′-CACCGTCA AGGCTGAGAAC-3′ and 5′-TGGTGAAGACGCCAGTGGA-3′).

### RNA preparation and transcriptome resequencing

TRIzol reagent (Invitrogen, Carlsbad, CA, USA) was used to extract the total RNA of SiHa-Vec (n = 3) and SiHa-Slug (n = 3) monoclonal cells in this study for transcriptome resequencing. The BGISEQ-500 platform was used to analyze the samples at the Beijing Genomics Institute (BGI), and the average output of each sample was 22.16 M. The average ratio of sample to genome was 94.15%, and the ratio of comparison to each gene set was 82.37%. A total of 17,838 genes were identified in this study. The experimental analysis used the NOISeq method, which is a novel nonparametric approach for the identification of differentially expressed genes (DEGs) based on the thresholds of log2-fold change > 1 and a probability ≥ 0.80, FDR ≤ 0.001. Subsequent data analysis was performed online by Dr. Tom from the Beijing Genomics Institute.

### Luciferase reporter assay

The promoter region of EpCAM was analyzed through the Jaspar online database () and UCSC Genome online database. Several alternative E-boxes were found in the proximal promoter of EpCAM: 171, -52, -981, and -1319 from the ATG site. The fragments containing such an E-box site were cloned into the pGL3-Basic vector (Promega, Madison, WI, USA) to generate EpCAM promoter reporter constructs. Plasmids (fragments of the EpCAM promoter region, firefly luciferase reporter and Renilla luciferase reporter) were cotransfected into tumor cells in triplicate using Lipofectamine 2000 (Invitrogen, Carlsbad, CA, USA). Firefly luciferase reporter and Renilla luciferase reporter activities were measured consecutively by using a dual luciferase assay kit (Promega, Madison, WI). Firefly luciferase activity was normalized to Renilla luciferase activity, and the experiment was performed in triplicate. The primers are listed in Additional file 1: Table S1. All constructs were verified by sequencing. The specific activity is shown as the fold change of the experimental group versus the control group.

### Chromatin immunoprecipitation

SiHa-Vec and SiHa-Slug cells were subjected to ChIP using the EZ-ChIP Assay kit (Millipore) following the manufacturer’s instructions. Ten micrograms of an anti-Slug antibody (#9585, Cell Signaling Technology) or 1 µg of mouse IgG was incubated with the chromatin fraction at 4 °C overnight. DNA was used for polymerase chain reaction amplification with EpCAM-specific primers, E-cadherin was used as a positive control and IgG was used as a negative control. The primers used for these studies are listed in Additional file 1: Table S2.

### Statistical analysis

All of statistical analysis in this study was performed with Graphpad Prism 8.0 software and SPSS software version 19.0. For comparison among groups, the one-way ANOVA was performed. A univariate analysis was analyzed by Student’s t-test (two-tailed) and the Mann–Whitney U-test, and presented as mean ± SD. The correlation among protein expression was detected by using Pearson correlation analysis. In all of the tests, statistical significance was defined as *p* < 0.05.

## Results

### Slug promotes the migratory and invasive abilities of cervical cancer cells ***in vitro***

**Fig. 1:**
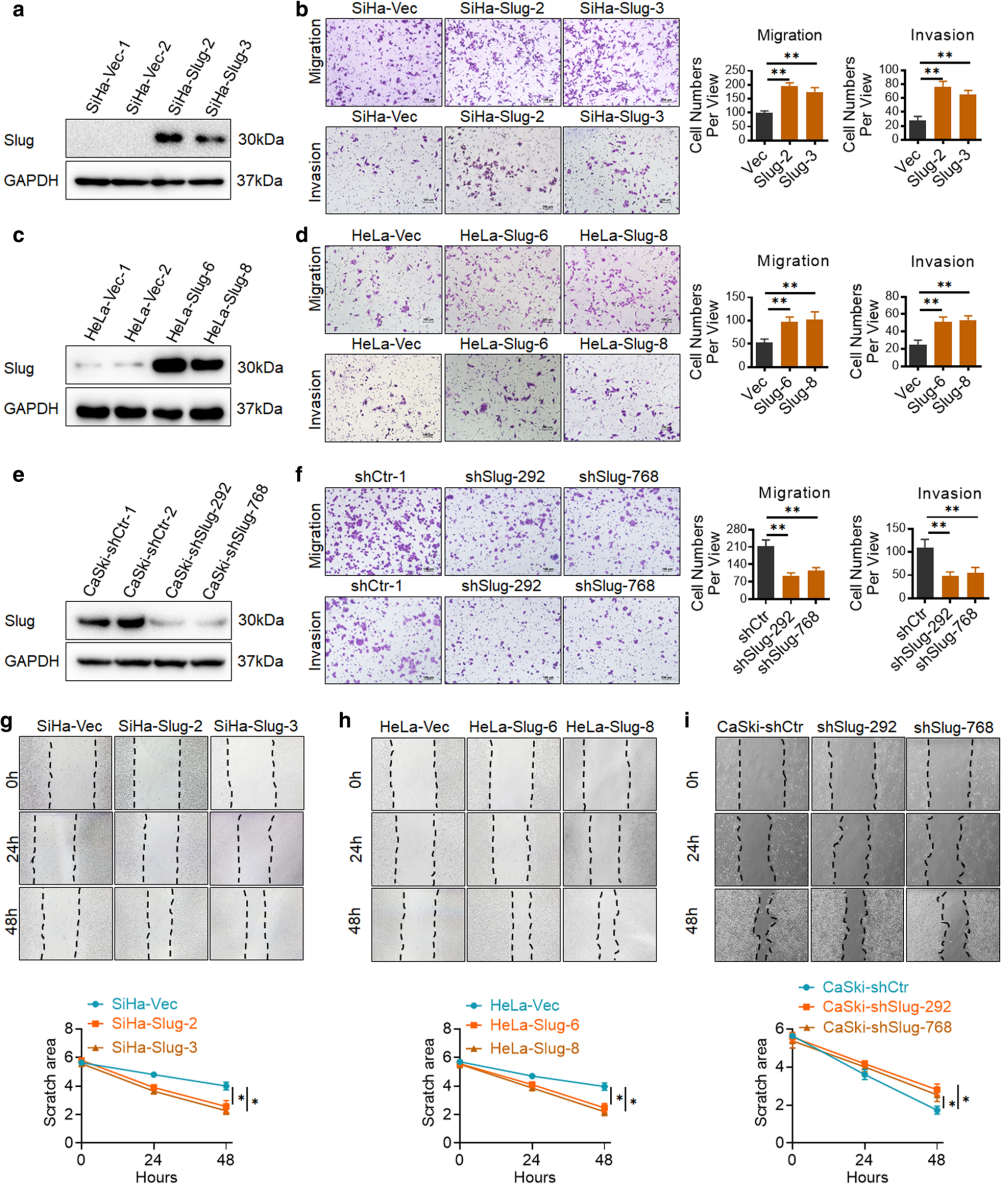
Slug promotes cell migration and invasion of cervical cancer cells* in vitro*. Slug-overexpressing (SiHa and HeLa) and Slug knockdown (CaSki) cells were identified by western blotting: **a** SiHa-Vec and SiHa-Slug cells; **c** HeLa-Vec and HeLa-Slug cells; and **e** CaSki-shControl and CaSki-shSlug cells. The migratory and invasive capacities of Slug-modified cells were analyzed by the transwell cell assay, and the number of migrated cells is shown (scale bar, 100 μm): **b** SiHa-Vec and SiHa-Slug cells; **d** HeLa-Vec and HeLa-Slug cells; and **f** CaSki-shControl and CaSki-shSlug cells. The migratory potential of Slug-modified cells was analyzed by wound-healing assays performed for 0, 24, and 48 h (scale bar, 200 μm): **g** SiHa-Vec and SiHa-Slug cells, and the quantitative analysis is shown; **h** HeLa-Vec and HeLa-Slug cells, and the quantitative analysis is shown; and **i** CaSki-shControl and CaSki-shSlug cells, and the quantitative analysis is shown

**Fig. 2:**
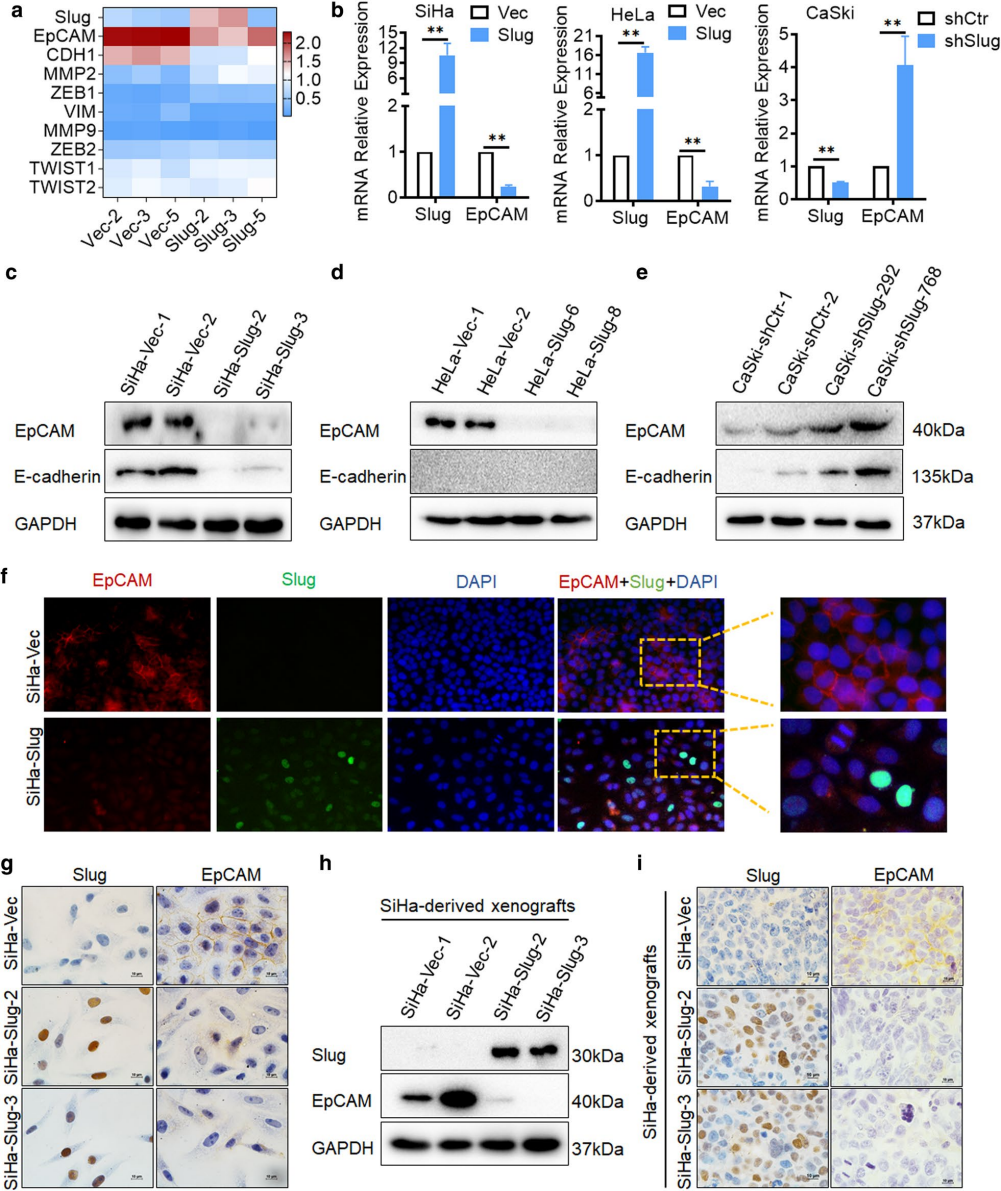
Slug suppresses EpCAM expression in cervical cancer cells. **a** Heatmap of the enriched differentially expressed genes identified by RNA transcriptome sequencing assays between the SiHa-Slug and SiHa-Vec groups: Slug, EpCAM, CDH1, MMP2, ZEB1, VIM, MMP9, ZEB2, TWIST1 and TWIST2; data were log^10^ normalized. **b** The mRNA level of EpCAM in Slug-modified cells was detected by real-time quantitative PCR, and the quantitative analysis is shown. The protein levels of EpCAM and E-cadherin were detected by western blotting: **c** SiHa-Vec and SiHa-Slug cells, **d** HeLa-Vec and HeLa-Slug cells, and **e** CaSki-shControl and CaSki-shSlug cells. The expression of Slug and EpCAM was detected in SiHa-Vec and SiHa-Slug cells by using immunofluorescence (**f**) and immunocytochemistry analyses (**g**), scale bars, 1000× and 400×. The expression of Slug and EpCAM was detected in mouse xenografted tumor tissues derived from SiHa-Vec and SiHa-Slug cells by using western blotting (**h**) and immunocytochemistry analysis (**i**), scale bars, 1000 ×. Data were statistically analyzed with Student’s t-test, and data are shown as the mean ± SD of three independent experiments. * *p* < 0.05 vs. control

To further investigate the function of Slug in the regulation of cell motility and distant metastasis in human cervical cancer cells, exogenous Slug was stably overexpressed by transfection of a Slug recombinant plasmid in SiHa cells (Fig. 1a, SiHa-Vec, SiHa-Slug-2 and SiHa-Slug3) and HeLa cells (Fig. 1c, HeLa-Vec, HeLa-Slug-6 and HeLa-Slug-8). Endogenous Slug was knocked down by using two of efficiently Slug shRNA vectors (shSlug-292 and shSlug-768) in CaSki cells (Fig. 1e, CaSki-shControl, CaSki-shSlug-292 and CaSki-shSlug-768). Then, transwell assays were performed to evaluate the capacity for motility in Slug-modified cervical cancer cells and control cells. As shown in Fig. 1b, after incubation for 48 h, the numbers of SiHa-Slug cells (195.20 ± 12.41 and 174.60 ± 15.26) that migrated across the uncoated membrane were much higher than those of SiHa-Vec cells (98.00 ± 8.43, *p* < 0.01). Similarly, the numbers of HeLa-Slug cells (97.60 ± 10.98 and 102.80 ± 16.35) that migrated across the uncoated membrane were much higher than those of HeLa-Vec cells (52.6 ± 7.85, *p* < 0.01, Fig. 1d). Conversely, when Slug expression was knocked down in CaSki cells by using the shRNA vector, the number of cells that migrated across the uncoated membrane was much lower in CaSki-shSlug cells (94.40 ± 11.30 and 113.20 ± 13.27) than in CaSki-shControl cells (211.80 ± 24.85, *p* < 0.01, Fig. 1f). Moreover, after incubation for 48 h, the wound was closed much faster in SiHa-Slug-2 and SiHa-Slug-3 cells (Fig. 1g, p < 0.01) and HeLa-Slug-6 and HeLa-Slug-8 cells (Fig. 2h, p < 0.01) than in their respective control cells (SiHa-Vec and HeLa-Vec cells, respectively). In addition, the wound was closed much slower in CaSki-shSlug-292 and CaSki-shSlug-768 cells (Fig. 1i, p < 0.01) than in CaSki-shControl cells. These results suggested that Slug promoted cell migration in cervical cancer cells *in vitro*.

Furthermore, transwell cambers were coated with Matrigel to detect changes in invasive capacity in Slug-modified cells. As shown in Fig. 1b, after incubation for 48 h, the numbers of SiHa-Slug cells (75.60 ± 8.56 and 64.60 ± 6.54) that migrated across the coated membrane were much higher than those of SiHa-Vec cells (27.60 ± 6.06, *p* < 0.01). Similarly, the numbers of HeLa-Slug cells (51.20 ± 5.68 and 54.60 ± 5.55) that migrated across the coated membrane were much higher than those of HeLa-Vec cells (25.20 ± 4.92, *p* < 0.05, Fig. 1d). Conversely, the number of cells that migrated across the coated membrane was much lower in the CaSki-shSlug group (48.40 ± 9.00 and 55.65 ± 11.77) than in the CaSki-shControl group (109.00 ± 17.99, *p* < 0.01, Fig. 1f). These results demonstrate that Slug enhances the invasive capacity of cervical cancer cells *in vitro*.

Interestingly, a lower immunoreactive score (IRS) of Slug was observed in 52 squamous cervical cancer (SCC) tissues in our previous study compared with 37 normal cervix tissues [21]. However, some SCC tissues exhibited high expression of Slug individually (Additional file 1: Figure S1G). Additionally, as the arrows marked in Additional file 1: Figure S1G indicate, several single cells with strong Slug staining were located in the outside of tumor areas (SCC08 and SCC25) or mixed with mesenchymal cells (SCC34 and SCC44). Although we could not affirm whether these Slug-stained cells were separated from the neighboring tumor area, it is possible that cells with high expression of Slug could endow migratory ability to cells and tended to be excluded from the tumor areas.

#### Slug inhibits EpCAM expression in cervical cancer cells

Slug is well known for its initiation of EMT by trans-suppressing E-cadherin in numerous cancers through its binding to the E-box in the proximal promoter region of the E-cadherin gene, further attenuating cell-cell adhesion [22, 23]. The trans-suppression effect of Slug on E-cadherin in cervical cancer cells was also confirmed in our previous study [21]. As expected, the protein level of E-cadherin was significantly decreased in Slug-overexpressing SiHa cells (Fig. 2c) and increased in Slug knockdown CaSki cells (Fig. 2e). However, E-cadherin was expressed at a very low protein level in HeLa cells (Fig. 2d), and this was also observed in our previous study [24]. Therefore, in addition to E-cadherin, Slug probably interacts with other molecules to regulate cell-cell adhesion and promote cell EMT in cervical cancer cells.

To further explore the potential molecules that participate in Slug-mediated EMT in cervical cancer cells, transcriptome sequencing analysis was performed in SiHa-Slug (n = 3) and SiHa-Vec (n = 3) monoclonal cell lines. As shown in Additional file 1: Figure S1A, a total of 17,838 transcripts were detected, and 500 upregulated and 294 downregulated genes were identified between the SiHa-Slug and SiHa-Vec groups. GO enrichment and KEGG pathway enrichment analyses revealed that Slug expression was associated with cell extracellular region, extracellular region part, proteinaceous extracellular matrix, ECM-receptor interaction and cell adhesion molecules (CAMs) (Additional file 1: Figure S1B). As expected, *CDH1 (E-cadherin)* expression was significantly decreased in Slug-overexpressing SiHa cells (Additional file 1: Figures S1E and S1F). Additionally, *epithelial cell adhesion molecule (EpCAM)*, a key cell-surface protein known to mediate cell–cell and cell–matrix interactions [25], was significantly decreased in Slug-overexpressing SiHa cells (Additional file 1: Figures S1C and S1D). Moreover, both the mRNA and protein levels of EpCAM in Slug-modified cells were confirmed by real-time PCR and western blotting. Consistent with the transcriptome sequencing analysis results, EpCAM was decreased in both SiHa-Slug and HeLa-Slug cells (Fig. 2b–d and Additional file 1: Figure S2, p < 0.05) and increased in Slug knockdown CaSki cells (Fig. 2b and e and Additional file 1: Figure S2, p < 0.05), either at the mRNA or protein level. Furthermore, immunofluorescence (Fig. 2f) and immunocytochemistry analyses (Fig. 2g and Additional file 1: Figure S2E) confirmed the reduction in EpCAM in SiHa-Slug cells. Additionally, the decreased protein level of EpCAM was confirmed in mouse xenografted tumor tissues derived from SiHa-Slug cells (the xenografted tumor tissues were collected in our previous study [21]) by western blot (Fig. 2h and Additional file 1: Figure S2D, p < 0.05) and immunohistochemistry (Fig. 2i and Additional file 1: Figure S2F, p < 0.05). All of these results indicated that Slug inhibited EpCAM expression in cervical cancer cells.

### Slug promotes distant metastasis in cervical cancer ***in vivo***

**Fig. 3:**
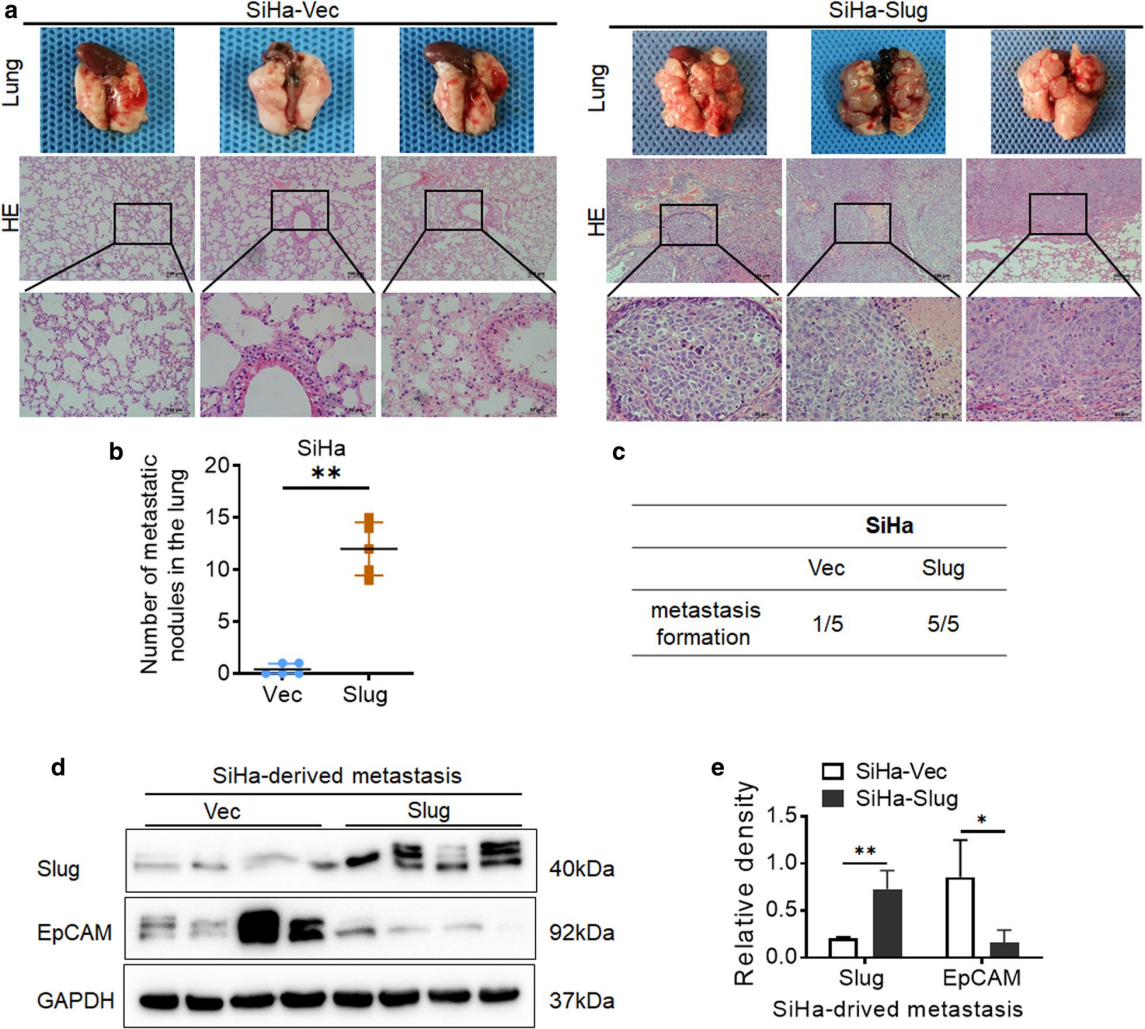
Slug promotes distant metastasis of SiHa and HeLa cells* in vivo*. **a** SiHa-Vec and SiHa-Slug cells were injected into female BALB/c nude mice via the tail vein. Representative hematoxylin and eosin-stained images of lung sections are presented to show the metastatic nodules in tumor lesions, scale bars, 500 μm and 200 μm. **b** The scatter plots show the number of metastatic nodules in the lungs as the mean ± SD (n = 5). **c** The number of mice that bore metastatic nodules. **d** The expression of Slug and EpCAM was detected in mouse metastasis tumor tissues derived from SiHa-Vec and SiHa-Slug cells by using western blotting, and the quantitative analysis was shown in (**e**). Data were statistically analyzed with Student’s t-test, and values are shown as the mean ± SD, * *p* < 0.05, ** *p* < 0.01 vs. control

To characterize the function of Slug in mediating distant metastasis of cervical cancer *in vivo*, 6 × 10^5^ SiHa-Vec and SiHa-Slug cells were injected into female nude mice via the tail vein. After two to three months, the lungs and livers of the tested mice were collected, metastatic nodules were quantified, and lung and liver sections were stained with hematoxylin and eosin (H&E). As shown in Fig. 3a–c, the mice bearing SiHa-Slug cells generated many more metastatic nodules in the lung organ than mice bearing SiHa-Vec cells. Regrettably, no metastatic nodules were observed in the liver in mice injected with SiHa-Slug cells or SiHa-Vec cells. This finding suggested that Slug promoted cell migration to distant sites and enhanced the metastatic potential of cervical cancer cells. Moreover, the decreased protein level of EpCAM was confirmed in mouse metastasis tumor tissues derived from SiHa-Slug cells by western blot (Fig. 3d and e, *p* < 0.05).

### Slug trans-suppresses EpCAM expression by binding to the E-box elements in the promoter region of EpCAM in cervical cancer cells

**Fig. 4:**
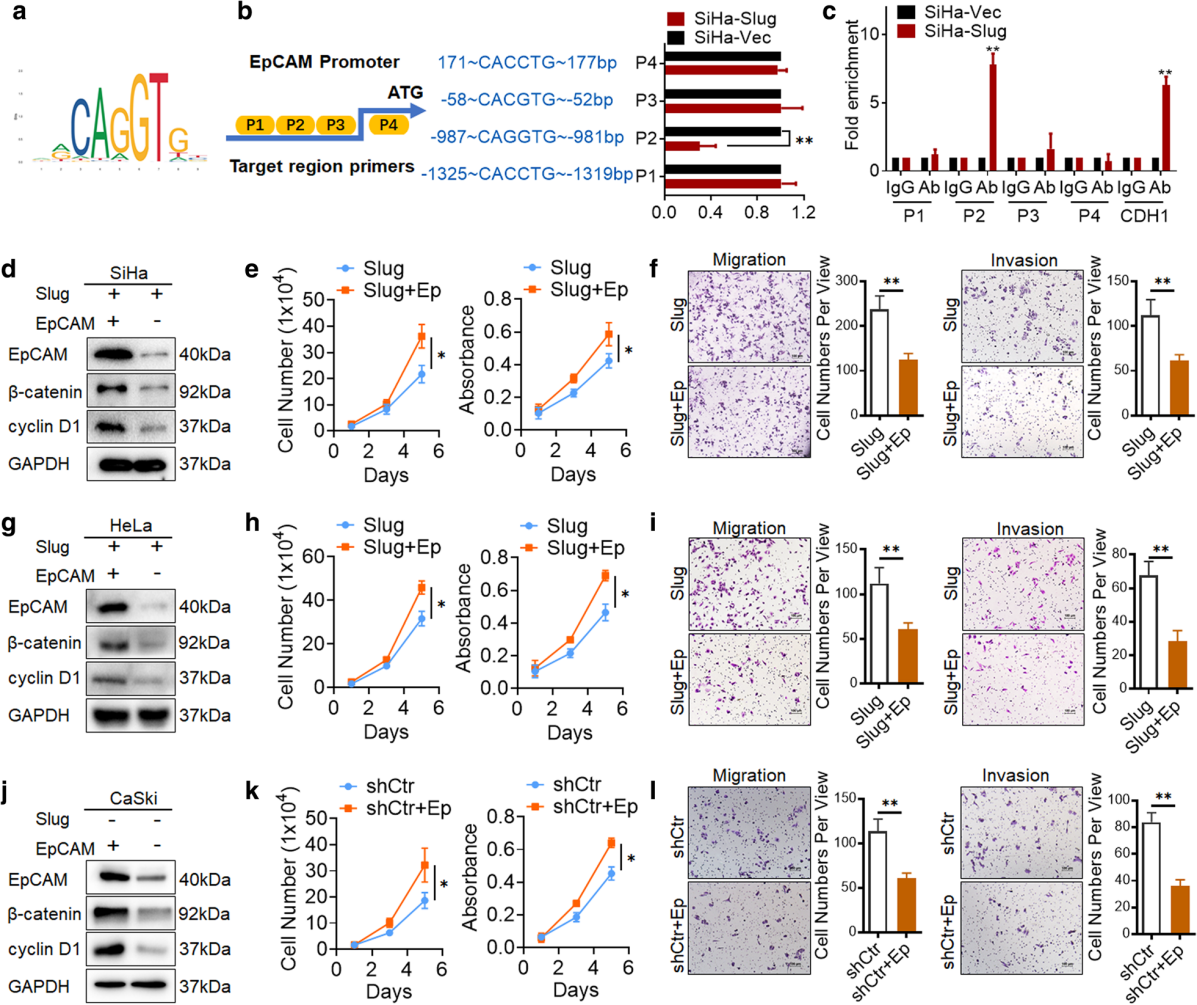
Rescuing EpCAM in Slug-overexpressing cells inhibits cell motility and promotes cell growth. **a** The potential binding site for Slug on the promoter region of EpCAM was analyzed through the Jaspar online database. **b** The activity of the EpCAM promoter was measured by using the dual luciferase assay and is presented as the fold change in the activity of SiHa-Slug cells versus SiHa-Vec cells. **c** A quantitative ChIP assay of the EpCAM promoter region in SiHa-Slug and SiHa-Vec cells is shown. The protein levels of EpCAM, β-catenin and cyclin D1 in Slug-overexpressing cells transiently transfected with an EpCAM recombinant plasmid were detected by western blotting: **d** SiHa-Slug cells, **g** HeLa-Slug cells, and **j **CaSki-shCtr cells. The growth potential of Slug-overexpressing cells by transient transfection with EpCAM recombinant plasmid was detected by generating growth curves and performing MTT assays: **e** SiHa-Slug cells, **h** HeLa-Slug cells and **k **CaSki-shCtr cells. The migratory and invasive capacities of Slug-overexpressing cells transiently transfected with an EpCAM recombinant plasmid were analyzed by the transwell cell migration assay: **f** SiHa-Slug cells, **i** HeLa-Slug cells and **l **CaSki-shCtr cells. Data were statistically analyzed with Student’s t-test, and data are shown as the mean ± SD of three independent experiments. * *p* < 0.05, ** *p* < 0.01 vs. control

As a transcriptional regulatory factor, Slug could recognize and directly bind the E-box motifs (CANNTG) in the proximal promoter region to trans-suppress the expression of target genes. Therefore, the promoter region of EpCAM was analyzed through the Jaspar online database () and UCSC Genome online database. Several alternative E-boxes were found in the proximal promoter of EpCAM: 171, -52, -981, and -1319 from the ATG site (Fig. 4a and b). A luciferase reporter assay was carried out to determine the transcriptional regulation activity of Slug for these alternative E-boxes on the EpCAM promotor. Truncated fragments containing the potential E-boxes in the EpCAM promoter region were transiently transfected into SiHa-Slug and SiHa-Vec cells. Slug overexpression resulted in a significant decrease in luciferase activity on the EpCAM promoter region in the upstream sequence site − 987 ~ CAGGTG~ − 981 (Fig. 4b, p < 0.05). Furthermore, a chromatin immunoprecipitation assay (ChIP) corroborated the interaction between Slug and the upstream sequence site of the EpCAM promoter (− 987 ~ CAGGTG~ − 981, Fig. 4c, p < 0.05), with the E-cadherin promoter region added as a positive control. Finally, these data identified an upstream sequence site of EpCAM (− 987 ~ CAGGTG~ − 981) that was described as a selected binding site for Slug to transcriptionally inhibit EpCAM in cervical cancer cells.

#### Rescuing EpCAM in slug‐overexpressing cells inhibits cell motility and promotes cell growth

To investigate whether the absence of EpCAM in Slug-overexpressing cells is involved in Slug mediating EMT and cell motility in cervical cancer cells, EpCAM expression was rescued via transient transfection of an EpCAM recombinant plasmid in Slug-overexpressing cells and CaSki-shCtr cells. Then, western blot analysis revealed the recovery of EpCAM expression in SiHa-Slug cells (Fig. 4d and S2G, p < 0.05), HeLa-Slug cells (Fig. 4g and Additional file 1: Figure S2H, p < 0.05) and CaSki-shCtr cells (Fig. 4j and Additional file 1: Figure S2I, p < 0.05), and an attenuated cell motility was observed in both SiHa-Slug cells (Fig. 4f, p < 0.05), HeLa-Slug cells (Fig. 4i, p < 0.05) and CaSki-shCtr cells (Fig. 4l, p < 0.05). On the other hand, EpCAM was reported to enhance cell cycle progression by upregulating cyclinD1 via the Wnt/β-catenin signaling pathway [26, 27]. As expected, the protein level of β-catenin and cyclin D1 was increased in EpCAM-expressing SiHa-Slug cells (Fig. 4d and Additional file 1: Figure S2G, p < 0.05), HeLa-Slug cells (Fig. 4g and Additional file 1: Figure S2H, p < 0.05) and CaSki-shCtr cells (Fig. 4j and Additional file 1: Figure S2I, p < 0.05), with a promotion of cell growth observed in both SiHa-Slug cells (Fig. 4e, p < 0.05), HeLa-Slug cells (Fig. 4h, p < 0.05) and CaSki-shCtr cells (Fig. 4k, p < 0.05). These results suggested that the presence of EpCAM in cervical cancer cells could promote cell growth but attenuate cell motility. The absence of EpCAM under Slug expression in cervical cancer cells is probably involved in Slug-regulated EMT and cell growth.

### Slug expression is correlated with EpCAM expression ***in vivo***

**Fig. 5:**
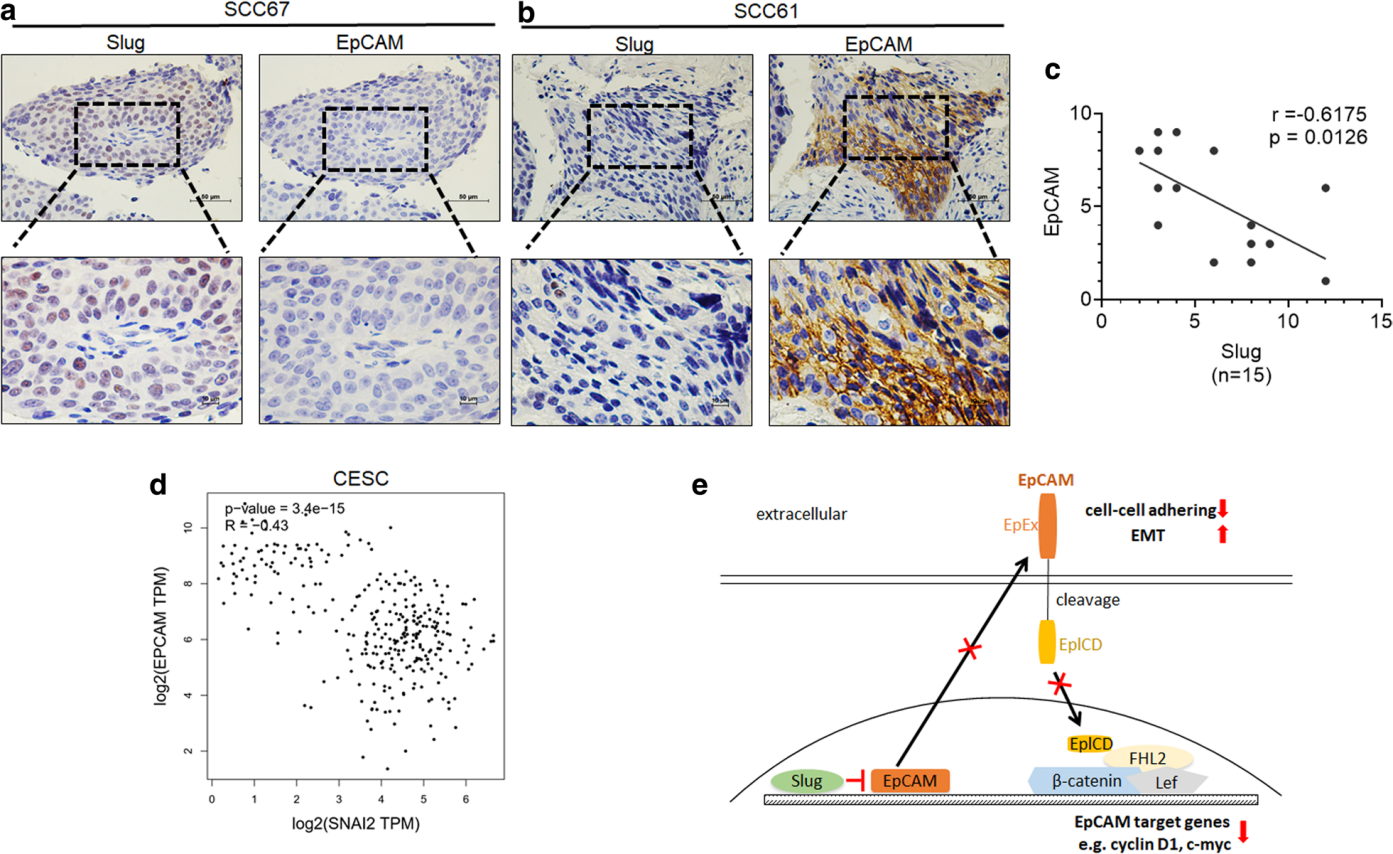
The correlation between the expression of Slug and EpCAM in human squamous cervical carcinoma (SCC) samples. **a** and **b** The expression of Slug and EpCAM was detected in serial sections of SCC samples by using immunocytochemistry analysis; scale bars, 400 × and 1000 ×. **c** The correlation between Slug and EpCAM in SCC samples was confirmed by using Pearson correlation analysis, n = 15. **d** The negative correlation between Slug and EpCAM expression in cervical squamous cell carcinoma and endocervical adenocarcinoma (CESC) was confirmed from the GEPIA online database. **e** Proposed model of the mechanisms by which Slug inhibits EpCAM expression and further promotes cell motility and inhibits cell growth in cervical cancer

As described above, the *in vitro* experiments revealed a negative correlation between the expression of Slug and EpCAM in cervical cancer cells. To validate these data *in vivo*, serial sections of human squamous cervical carcinoma (SCC) samples (n = 15) were immunostained with antibodies specific for Slug and EpCAM. Furthermore, the negative correlation between Slug and EpCAM expression in these SCC samples was confirmed by using Pearson correlation analysis (Fig. 5c, r = − 0.6159, *p* = 0.0145). Additionally, the negative correlation between Slug and EpCAM expression in cervical squamous cell carcinoma and endocervical adenocarcinoma (CESC) was confirmed from the GEPIA online database (Fig. 5d). Additionally, the negative correlation between Slug and EpCAM expression in bladder urothelial carcinoma (BLCA), colon adenocarcinoma (COAD), esophageal carcinoma (ESCA), breast invasive carcinoma (BRCA), head and neck squamous cell carcinoma (HNSC), lung adenocarcinoma (LUAD), lung squamous cell carcinoma (LUSC), prostate adenocarcinoma (PRAD) and stomach adenocarcinoma (STAD) was confirmed from the GEPIA online database (Additional file 1: Figure S3A). Although several interesting expression patterns and a negative correlation between Slug and EpCAM were observed, only nine samples were analyzed in this study. Therefore, more cases still need to be analyzed to further confirm the negative correlation between Slug and EpCAM in cervical cancer.

## Discussion

Slug is a key regulator for initiating EMT either in normal or malignant cells and is involved in embryonic development and facilitates distant metastasis of cancer cells. Several studies have reported the positive effect of Slug on initiating cell EMT and promoting cell motility by trans-suppressing E-cadherin in cervical cancer. When Slug was reduced by TRIM62 (tripartite motif containing 62) [28] and DDX3 (DEAD box polypeptide 3) [29] in cervical cancer cells, E-cadherin mRNA and protein expression levels were significantly increased in these cells, further suppressing epithelial-mesenchymal transition (EMT) and inhibiting their migration and invasion abilities. Conversely, Slug expression could be stimulated by long noncoding RNA-SNHG12 [7], LIV-1 (estrogen-regulated zinc transporter) [30] or long noncoding RNA-CC3 [6] in cervical cancer, further reducing E-cadherin expression and subsequently facilitating cell EMT and promoting distant metastasis. Additionally, this promotion of cell motility and tumor metastasis in cervical cancer was reconfirmed in this study. Exogenously expressed Slug in HeLa and SiHa cells significantly enhanced cell motility *in vitro* and promoted distant metastasis *in vivo*. The trans-suppression effect of Slug on E-cadherin was also observed in SiHa cells. However, surprisingly, E-cadherin was expressed at a very low protein level in HeLa cells, but exogenously expressed Slug in HeLa cells still has the capacity to enhance cell migratory and invasive abilities *in vitro*. Therefore, we hypothesized that there is probably an E-cadherin-independent mechanism by which Slug initiates EMT in cervical cancer cells.

Transcriptome sequencing analysis was performed to identify the potential factors that are probably involved in Slug-mediated EMT in cervical cancer cells. Several classical EMT-related molecules were identified by transcriptome sequencing analysis, including E-cadherin, MMP2 and ZEB1. Unfortunately, MMP9, vimentin and Twist levels were not significantly different between the SiHa-Slug and SiHa-Vec groups. GO enrichment analysis revealed that exogenously expressed Slug in SiHa cell was associated with cell junctions, cell extracellular regions, extracellular region parts and proteinaceous extracellular matrix. Surprisingly, *epithelial cell adhesion molecule (EpCAM)*, a key cell-surface protein known to mediate cell–cell junction and cell–matrix interactions [25], was significantly decreased in the SiHa-Slug group. The reduction in EpCAM in Slug-overexpressing cells was confirmed by both *in vitro* and *in vivo* experiments. Further luciferase reporter and chromatin immunoprecipitation assays corroborated the trans-suppression effect of Slug on EpCAM in cervical cancer cells via its binding to an alternative sequence site upstream of the EpCAM promoter. Moreover, a negative correlation between Slug and EpCAM was observed in human squamous cervical carcinoma (SCC) samples and further confirmed in the GEPIA online database. All of these *in vitro* and *in vivo* experiments demonstrated that Slug could trans-suppress EpCAM expression in cervical cancer cells.

Slug is well known to play a vital role in initiating EMT and promoting distant metastasis in numerous cancers, including cervical cancer [23]. However, as described above, EpCAM is the most puzzling due to its biphasic effects on the regulation of cell-cell adhesion and EMT during metastasis progression in different types of cancers, and the function of EpCAM in regulating cell motility in cervical cancer remains unclear. Therefore, whether the absence of EpCAM in cervical cancer cells that resulted from Slug overexpression promotes or inhibits EMT and cell motility, is still unclear. Accordingly, EpCAM expression was rescued by transiently transfecting an EpCAM recombinant plasmid in Slug-overexpressing SiHa and HeLa cells, which then exhibited attenuated cell migration and invasion *in vitro*, suggesting that the presence of EpCAM in cervical cancer cells is probably disadvantageous for cell motility. On the other hand, EpCAM has been reported to promote cell growth by regulating classical cyclins (e.g., cyclin D1, cyclin A and cyclin E) via the Wnt/β-catenin signaling pathway [26, 27]. Coincidentally, attenuation of the Wnt/β-catenin signaling pathway and a reduction of cyclin D1 were observed in Slug-overexpressing cells in our previous study [21]. Therefore, the protein level of cyclin D1 was detected in cells with restored EpCAM expression. After transient transfection of an EpCAM recombinant plasmid in Slug-overexpressing SiHa and HeLa cells, the cells exhibited an induction of cyclin D1 and promotion of cell growth. All of these results suggest that the presence of EpCAM in cervical cancer cells could promote cell proliferation but attenuate cell motility. The absence of EpCAM under Slug expression in cervical cancer cells is probably involved in Slug-regulated EMT.

In conclusion, our study further confirmed the positive effect of Slug on regulating cell motility and promoting metastasis in cervical cancer. In addition, this study revealed a trans-suppression effect of Slug on EpCAM through its binding of the proximal promoter region of EpCAM in cervical cancer cells. This absence of EpCAM under Slug expression in cervical cancer cells probably inhibited cell-cell adhesion and participated in Slug-initiated EMT progression, further enhancing tumor metastasis. These results support a potential alternative mechanism by which Slug promotes cell motility in cervical cancer in addition to its trans-suppression effect on E-cadherin.

The negative correlation between Slug and EpCAM expression in cervical cancer was confirmed by both *in vitro* and *in vivo* experiments in this study. However, on account of the small number of human squamous cervical carcinoma samples (total of 15) used in this study, it was difficult to fully reflect the real interactive regulation between Slug and EpCAM during the whole progression from the initiation of EMT in the primary site to the formation of metastatic tumors in distant organs. Additionally, the full-length EpCAM protein can be divided into several essential parts, including the EpCAM cleaved extracellular domain (EpEX) and EpCAM cleaved intracellular domain (EpICD) [10]. Accumulated studies on the promotion of EMT by different parts of the EpCAM protein in numerous cancers should also be considered [25]. There might be a window or stage that requires different functions of EpCAM during the process of EMT in cancer cells to further participate in the metastasis process. The function of EpCAM and the interactive regulation between Slug and EpCAM during cells undergoing EMT to form metastatic tumors in cervical cancer still need more research to be further clarified.

## Supplementary Information

**Additional file 1: Figure S1.** The transcriptome sequencing analysis in SiHa-Slug and SiHa-Vec cells. (A) Total of 500 up-regulated and 294 downregulated genes was identified between SiHa-Slug and SiHa-Vec groups (n=3) by using the transcriptome sequencing analysis, and shown with Volcano Plot. (B) Gene Ontology (GO) enrichment analysis and KEGG Pathway enrichment analysis identified the alteration of cellular function between SiHa-Slug and SiHa-Vec groups. (C) and (D) The expression of EpCAM in SiHa-Slug and SiHa-Vec cell lines. (E) and (F) The expression of EpCAM in SiHa-Slug and SiHa-Vec cell lines. (G) Immunohistochemistry (IHC) was performed to detect Slug expression in human squamous cervical carcinoma (SCC) samples at 1000×. The tumor area is marked by the dotted line, and the single cells with strong Slug staining adjacent to the tumor area are marked by the arrows. Data were statistically analyzed with Student’s t-test, and data are shown as the mean±SD of three independent experiments. * P<0.05, ** P<0.01 vs. control. **Figure S2.** The quantitative analysis for western blot, immunohistochemical and immunocychemistry stains. The quantitative analysis for western blot of EpCAM and CDH1 in Slug-modified cells: (A) SiHa-Vec and SiHa-Slug cells; (B) HeLa-Vec and HeLa-Slug cells; (C) CaSki-shControl and CaSki-shSlug cells. (D) The quantitative analysis for western blot of EpCAM and Slug in mouse xenografted tumor tissues that derived from SiHa-Vec and SiHa-Slug cells. (E) The quantitative analysis for immunohistochemical stains of EpCAM and Slug in SiHa-Vec and SiHa-Slug cells. (F) The quantitative analysis for immunohistochemical stains of EpCAM and Slug in mouse xenografted tumor tissues that derived from SiHa-Vec and SiHa-Slug cells. (G) The quantitative analysis for western blot of EpCAM, β-catenin and cyclin D1 in SiHa-Slug cells by transiently transfecting with an EpCAM recombinant plasmid. (H) The quantitative analysis for western blot of EpCAM, β-catenin and cyclin D1 in HeLa-Slug cells by transiently transfecting with an EpCAM recombinant plasmid. (I) The quantitative analysis for western blot of EpCAM, β-catenin and cyclin D1 in shCaSki-Ctr cells by transiently transfecting with an EpCAM recombinant plasmid. Data were statistically analyzed with Student’s t-test, data are shown as the mean±SD of three independent experiments. * p<0.05, ** p<0.01 vs. control. **Figure S3.** The negative correlation between Slug expression and EpCAM in tumors analyzed from GEPIA online database. (A) The negative correlation between Slug expression and EpCAM in bladder Urothelial Carcinoma (BLCA), breast invasive carcinoma (BRCA), colon adenocarcinoma (COAD), esophageal carcinoma (ESCA), head and neck squamous cell carcinoma (HNSC), lung adenocarcinoma (LUAD), lung squamous cell carcinoma (LUSC), prostate adenocarcinoma (PRAD) and stomach adenocarcinoma (STAD) was analyzed by using Pearson correlation analysis from GEPIA online database. (B) The western blotting for Slug of ChIP analysis. **Table S1.** The list of primer sequences that used for luciferase assays in this study.** Table S2.** The list of primer sequences that used for chromatin immunoprecipitation assay (ChIP) in this study.

## Data Availability

The transcriptomic dataset generated and analyzed during the current study are available in the NCBI SRP repository, : PRJNA682718.

## Abbreviations

EpCAM: Epithelial cell adhesion molecule
SCC: Squamous cervical carcinoma
EMT: Epithelial-mesenchymal transition
Daxx: Death domain-associated protein
USP5: Ubiquitin-specific protease 5
TNFα: Tumor necrosis factor-α
TGF-β1: Transforming growth factor-β1
ASC: Cervical adenosquamous carcinoma
ATCC: American Type Culture Collection
DMEM: Dulbecco’s modified Eagle’s medium
IHC: Immunohistochemistry
SFP: Specific pathogen-free
ChIP: Chromatin immunoprecipitation
IRS: Immunoreactive score
H&E: Hematoxylin and eosin
CESC: Cervical squamous cell carcinoma and endocervical adenocarcinoma
BLCA: Bladder urothelial carcinoma
COAD: Colon adenocarcinoma
ESCA: Esophageal carcinoma
BRCA: Breast invasive carcinoma
HNSC: Head and neck squamous cell carcinoma
LUAD: Lung adenocarcinoma
LUSC: Lung squamous cell carcinoma
PRAD: Prostate adenocarcinoma
STAD: Stomach adenocarcinoma
TRIM62: Tripartite motif containing 62
DDX3: DEAD box polypeptide 3
EpEX: EpCAM cleaved extracellular domain
EpICD: EpCAM cleaved intracellular domain
