# Correction: Absence of EpCAM in cervical cancer cells is involved in slug induced epithelial-mesenchymal transition

**DOI:** 10.1186/s12935-025-03706-0

**Published:** 2025-03-15

**Authors:** Xian Liu, Qian Feng, Yanru Zhang, PengSheng Zheng, Nan Cui

**Affiliations:** 1 Department of Reproductive Medicine, The First Affiliated Hospital of the Medical College, Xi’an Jiaotong University, 76 West Yanta Road, Shaanxi Province Xi’an, 710061 People’s Republic of China; 2 Section of Cancer Stem Cell Research, Key Laboratory of Environment and Genes Related to Diseases, Ministry of Education of the People’s Republic of China, Shaanxi Province Xi’an, 710061 People’s Republic of China

**Correction to**: **Cancer Cell Int (2021) 21:163**

10.1186/s12935-021-01858-3

In this article [1], there was an error in Fig. 1D, where the graph depicting HeLa-Slug-8 (invasion) contains inaccuracies due to an inadvertent omission in the data presentation. For completeness and transparency, the old incorrect Fig. 1 and the correct Fig. 1 are displayed below.

Incorrect Fig. 1.

**Fig. 1:**
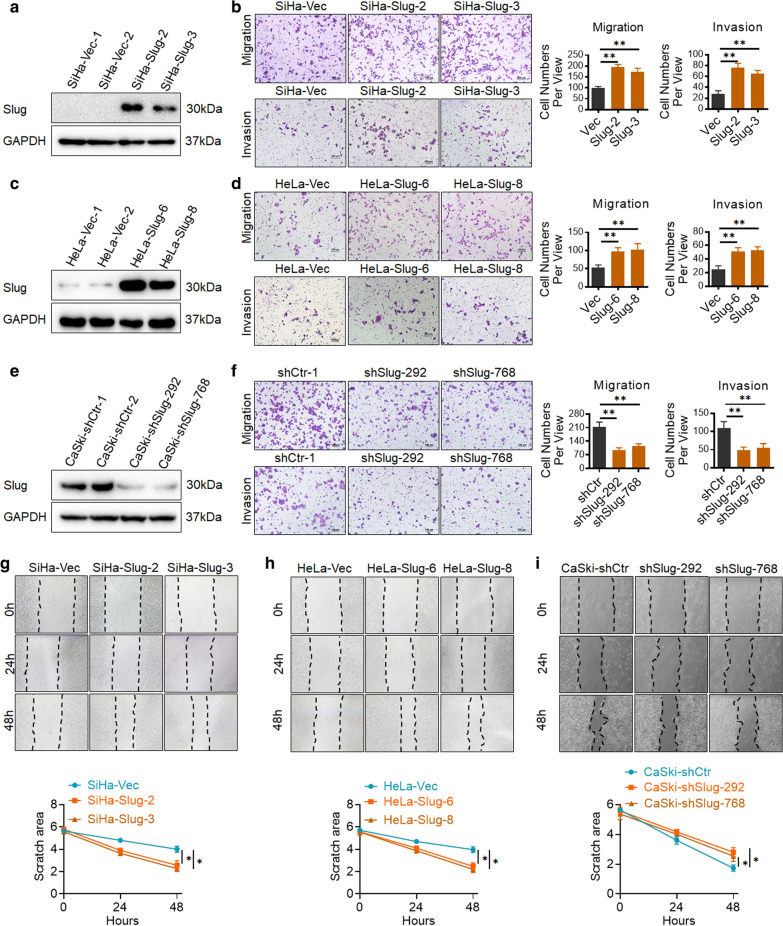
Slug promotes cell migration and invasion of cervical cancer cells in vitro. Slug-overexpressing (SiHa and HeLa) and Slug knockdown (CaSki) cells were identified by western blotting: **a** SiHa-Vec and SiHa-Slug cells; **c** HeLa-Vec and HeLa-Slug cells; and **e** CaSki-shControl and CaSki-shSlug cells. The migratory and invasive capacities of Slug-modified cells were analyzed by the transwell cell assay, and the number of migrated cells is shown (scale bar, 100 μm): **b** SiHa-Vec and SiHa-Slug cells; **d** HeLa-Vec and HeLa-Slug cells; and **f** CaSki-shControl and CaSki-shSlug cells. The migratory potential of Slug-modified cells was analyzed by wound-healing assays performed for 0, 24, and 48 h (scale bar, 200 μm): **g** SiHa-Vec and SiHa-Slug cells, and the quantitative analysis is shown; **h** HeLa-Vec and HeLa-Slug cells, and the quantitative analysis is shown; and **i** CaSki-shControl and CaSki-shSlug cells, and the quantitative analysis is shown

Correct Fig. 1.

**Fig. 1:**
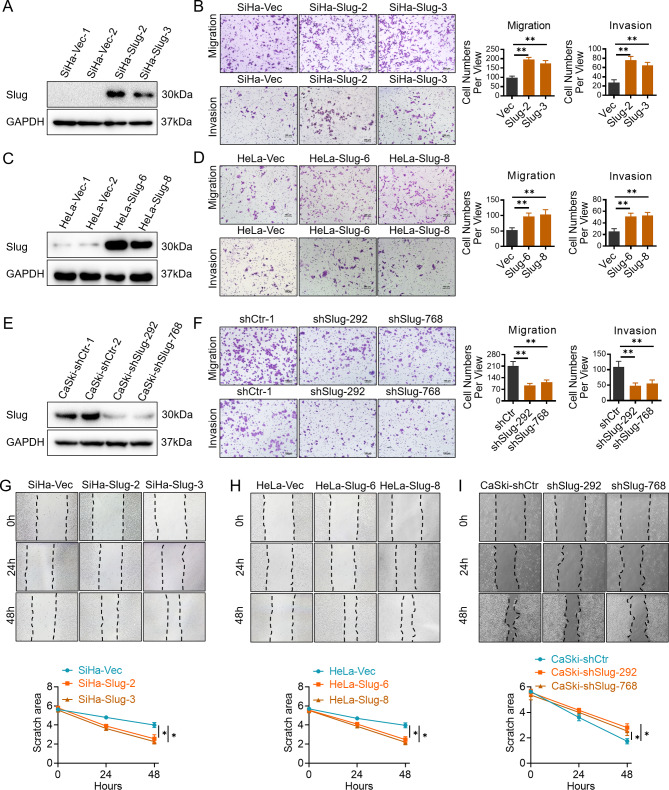
Slug promotes cell migration and invasion of cervical cancer cells in vitro. Slug-overexpressing (SiHa and HeLa) and Slug knockdown (CaSki) cells were identified by western blotting: **a** SiHa-Vec and SiHa-Slug cells; **c** HeLa-Vec and HeLa-Slug cells; and **e** CaSki-shControl and CaSki-shSlug cells. The migratory and invasive capacities of Slug-modified cells were analyzed by the transwell cell assay, and the number of migrated cells is shown (scale bar, 100 μm): **b** SiHa-Vec and SiHa-Slug cells; **d** HeLa-Vec and HeLa-Slug cells; and **f** CaSki-shControl and CaSki-shSlug cells. The migratory potential of Slug-modified cells was analyzed by wound-healing assays performed for 0, 24, and 48 h (scale bar, 200 μm): **g** SiHa-Vec and SiHa-Slug cells, and the quantitative analysis is shown; **h** HeLa-Vec and HeLa-Slug cells, and the quantitative analysis is shown; and **i** CaSki-shControl and CaSki-shSlug cells, and the quantitative analysis is shown
