# Supplementary material for: Absence of EpCAM in cervical cancer cells is involved in slug induced epithelial-mesenchymal transition
Source: Cancer Cell Int. 2021 Mar 10;21:163. doi: 10.1186/s12935-021-01858-3 (PMC7944906; doi:10.1186/s12935-021-01858-3)
Supplement: Supplementary file 1 — Additional file 1: Figure S1. The transcriptome sequencing analysis in SiHa-Slug and SiHa-Vec cells. (A) Total of 500 up-regulated and 294 downregulated genes was identified between SiHa-Slug and SiHa-Vec groups (n=3) by using the transcriptome sequencing analysis, and shown with Volcano Plot. (B) Gene Ontology (GO) enrichment analysis and KEGG Pathway enrichment analysis identified the alteration of cellular function between SiHa-Slug and SiHa-Vec groups. (C) and (D) The expression of EpCAM in SiHa-Slug and SiHa-Vec cell lines. (E) and (F) The expression of EpCAM in SiHa-Slug and SiHa-Vec cell lines. (G) Immunohistochemistry (IHC) was performed to detect Slug expression in human squamous cervical carcinoma (SCC) samples at 1000×. The tumor area is marked by the dotted line, and the single cells with strong Slug staining adjacent to the tumor area are marked by the arrows. Data were statistically analyzed with Student’s t-test, and data are shown as the mean±SD of three independent experiments. * P<0.05, ** P<0.01 vs. control. Figure S2. The quantitative analysis for western blot, immunohistochemical and immunocychemistry stains. The quantitative analysis for western blot of EpCAM and CDH1 in Slug-modified cells: (A) SiHa-Vec and SiHa-Slug cells; (B) HeLa-Vec and HeLa-Slug cells; (C) CaSki-shControl and CaSki-shSlug cells. (D) The quantitative analysis for western blot of EpCAM and Slug in mouse xenografted tumor tissues that derived from SiHa-Vec and SiHa-Slug cells. (E) The quantitative analysis for immunohistochemical stains of EpCAM and Slug in SiHa-Vec and SiHa-Slug cells. (F) The quantitative analysis for immunohistochemical stains of EpCAM and Slug in mouse xenografted tumor tissues that derived from SiHa-Vec and SiHa-Slug cells. (G) The quantitative analysis for western blot of EpCAM, β-catenin and cyclin D1 in SiHa-Slug cells by transiently transfecting with an EpCAM recombinant plasmid. (H) The quantitative analysis for western blot of EpCAM, β-catenin and [file 12935_2021_1858_MOESM1_ESM.doc]

**Supplement Information**

**
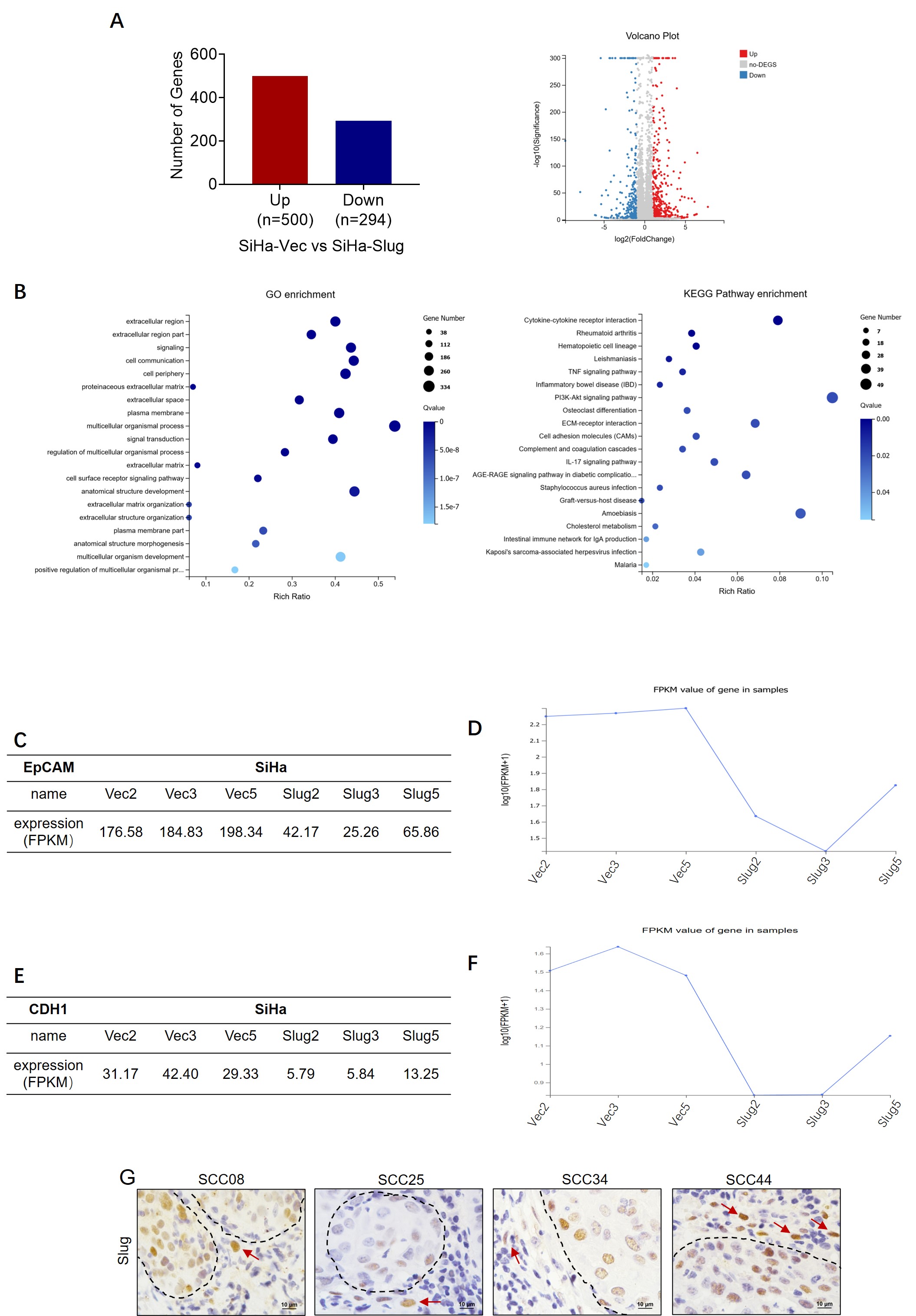
**

**Figure S1: The transcriptome sequencing analysis in SiHa-Slug and SiHa-Vec cells.**

(A) Total of 500 up-regulated and 294 downregulated genes was identiﬁed between SiHa-Slug and SiHa-Vec groups (n=3) by using the transcriptome sequencing analysis, and shown with Volcano Plot. (B) Gene Ontology (GO) enrichment analysis and KEGG Pathway enrichment analysis identiﬁed the alteration of cellular function between SiHa-Slug and SiHa-Vec groups. (C) and (D) The expression of EpCAM in SiHa-Slug and SiHa-Vec cell lines. (E) and (F) The expression of EpCAM in SiHa-Slug and SiHa-Vec cell lines. (G) Immunohistochemistry (IHC) was performed to detect Slug expression in human squamous cervical carcinoma (SCC) samples at 1000×. The tumor area is marked by the dotted line, and the single cells with strong Slug staining adjacent to the tumor area are marked by the arrows. Data were statistically analyzed with Student’s t-test, and data are shown as the mean±SD of three independent experiments. * P<0.05, ** P<0.01 vs. control.

**
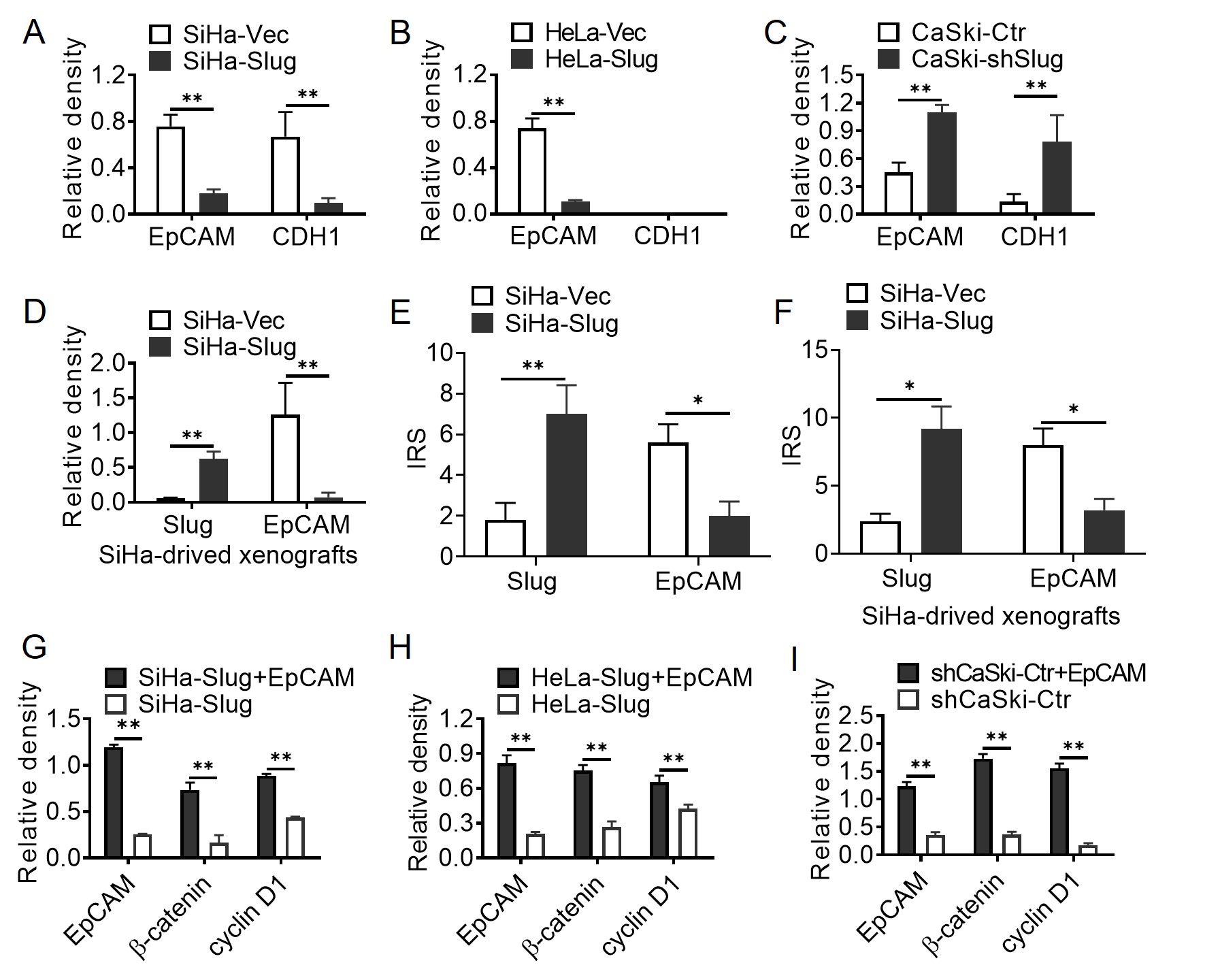
**

**Figure S2: The quantitative analysis for western blot, immunohistochemical and immunocychemistry stains**

The quantitative analysis for western blot of EpCAM and CDH1 in Slug-modified cells: (A) SiHa-Vec and SiHa-Slug cells; (B) HeLa-Vec and HeLa-Slug cells; (C) CaSki-shControl and CaSki-shSlug cells. (D) The quantitative analysis for western blot of EpCAM and Slug in mouse xenografted tumor tissues that derived from SiHa-Vec and SiHa-Slug cells. (E) The quantitative analysis for immunohistochemical stains of EpCAM and Slug in SiHa-Vec and SiHa-Slug cells. (F) The quantitative analysis for immunohistochemical stains of EpCAM and Slug in mouse xenografted tumor tissues that derived from SiHa-Vec and SiHa-Slug cells. (G) The quantitative analysis for western blot of EpCAM, β-catenin and cyclin D1 in SiHa-Slug cells by transiently transfecting with an EpCAM recombinant plasmid. (H) The quantitative analysis for western blot of EpCAM, β-catenin and cyclin D1 in HeLa-Slug cells by transiently transfecting with an EpCAM recombinant plasmid. (I) The quantitative analysis for western blot of EpCAM, β-catenin and cyclin D1 in shCaSki-Ctr cells by transiently transfecting with an EpCAM recombinant plasmid. Data were statistically analyzed with Student’s t-test, data are shown as the mean±SD of three independent experiments. * *p*<0.05, ** *p*<0.01 vs. control.


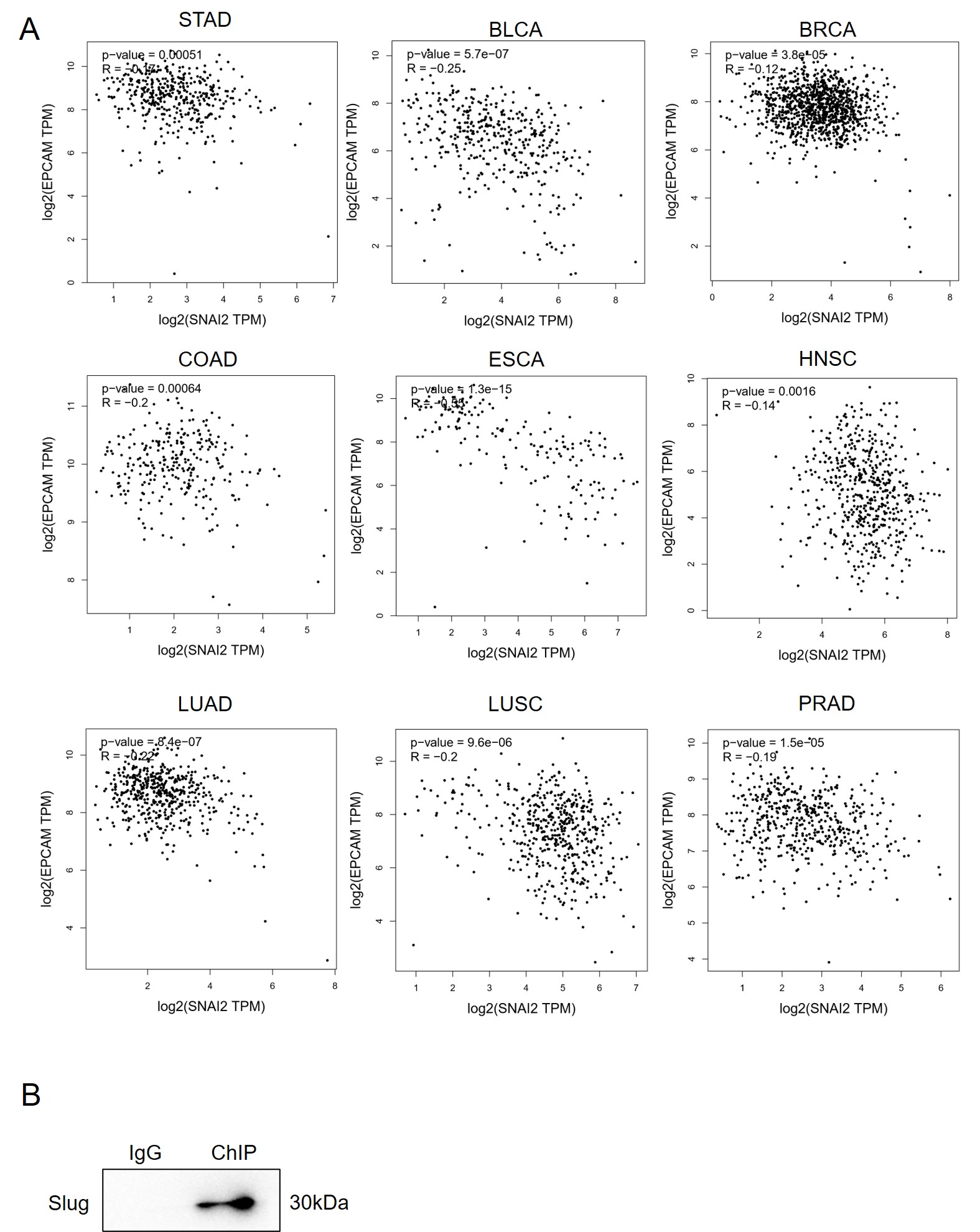


**Figure S3: The negative correlation between Slug expression and EpCAM in tumors analyzed from GEPIA online database.**

(A) The negative correlation between Slug expression and EpCAM in bladder Urothelial Carcinoma (BLCA), breast invasive carcinoma (BRCA), colon adenocarcinoma (COAD), esophageal carcinoma (ESCA), head and neck squamous cell carcinoma (HNSC), lung adenocarcinoma (LUAD), lung squamous cell carcinoma (LUSC), prostate adenocarcinoma (PRAD) and stomach adenocarcinoma (STAD) was analyzed by using Pearson correlation analysis from GEPIA online database. (B) The western blotting for Slug of ChIP analysis.

**Table S1. The list of primer sequences that used for luciferase assays in this study.**

| **Primer name** | **Location (bp)** | **F/R** | **Sequence** |
| --- | --- | --- | --- |
| **P1** | -1325～-1319 | F | GAAGATCTTCTTAAATATTTGTCTGTGG |
|  |  | R | CGACGCGTAGTAGAGACGAGGTT |
| **P2** | -987~-981 | F | GAAGATCTTATTTATCTGGCACCT |
|  |  | R | CGACGCGTGGTATTAAAACTTG |
| **P3** | -58~-52 | F | GAAGATCTGAAATACTAAGAATCCAGG |
|  |  | R | CGACGCGTTCCTTTGTCTCCAG |
| **P4** | 171~177 | F | GAAGATCTGCTGTAAAAGGACTAAGTAAGC |
|  |  | R | CGACGCGTGTTGTGAAAGCCTT |

**Table S2. The list of primer sequences that used for chromatin immunoprecipitation assay (ChIP) in this study.**

| **Primer name** | **Location** | **F/R** | **Sequence** |
| --- | --- | --- | --- |
| **P1** | -534~-462 | F | GCAGTGCTCGTGCCTGTAAT |
|  |  | R | CATGATGGCCAGGCTGGT |
| **P2** | -738~-644 | F | TATTTATCTGGCACCTAC |
|  |  | R | CTGGGTAATATTTTATAGG |
| **P3** | -824~-717 | F | AGGGGACTGCCAGGAACAC |
|  |  | R | CCTCTCCTTTGTCTCCAGGC |
| **P4** | -876~-788 | F | AAGTAAGCAAAGGTGCCTGT |
|  |  | R | TACGCTTTGCTCTCAGTGTG |
| **E-cadherin** | -25~110 | F | CGTCGGAACTGCAAAGC |
|  |  | R | TATGTGCGGTCGGGTCG |
| **3’UTR** |  | F | TTGAAGATTATAGAAGAAGG |
|  |  | R | TAACAAACTCATGACCTTC |
